# A Smartphone App to Support Carers of People Living With Cancer: A Feasibility and Usability Study

**DOI:** 10.2196/11779

**Published:** 2019-01-31

**Authors:** Natalie Heynsbergh, Leila Heckel, Mari Botti, Patricia M Livingston

**Affiliations:** 1 School of Nursing and Midwifery Faculty of Health Deakin University Geelong Australia; 2 Epworth HealthCare Melbourne Australia; 3 Faculty of Health Deakin University Geelong Australia

**Keywords:** cancer, carer, mobile app, smartphone, technology, mobile phone

## Abstract

**Background:**

Carers experience unique needs while caring for someone with cancer. Interventions that address carers’ needs and well-being have been developed and tested; however, the use of smartphone apps to support adult carers looking after another adult with cancer has not been assessed.

**Objective:**

The objective of this study was to test the feasibility, usability, and acceptability of a smartphone app, called the Carer Guide App, for carers of people with colorectal cancer.

**Methods:**

We recruited carers of people with colorectal cancer from outpatient day oncology units and provided them with access to the smartphone app for 30 days. Carers had access to video instructions and email contact details for technical support. Carers received 2 email messages per week that directed them to resources available within the app. Carers completed demographic questions at baseline and questions related to feasibility and usability at 30 days post app download. We used recruitment and attrition rates to determine feasibility and relevance of content to carers’ needs as self-reported by carers. We assessed usability through the ease of navigation and design and use of technical support or instructional videos. Acceptability was measured through self-reported usage, usage statistics provided by Google Analytics, and comments for improvement.

**Results:**

We recruited 31% (26/85) eligible carers into the trial. Of the 26 carers, the majority were female (19, 73%), on average 57 years of age, were caring for a spouse with cancer (19, 73%), and held a university degree (19, 73%). Regarding feasibility, carers perceived the content of the Carer Guide App as relevant to the information they were seeking. Regarding usability, carers perceived the navigation and design of the app as easy to use. Of the 26 carers, 4 (15%) viewed the downloading and navigation video and 7 (27%) used the contact email address for queries and comments. Acceptability: On average, carers used the smartphone app for 22 minutes (SD 21 minutes) over the 30-day trial. Of 26 participants, 19 completed a follow-up questionnaire. Of 19 carers, 7 (37%) logged on 3 to 4 times during the 30 days and 5 (26%) logged on more than 5 times. The majority (16/19, 84%) of carers stated that they would recommend the app be available for all carers. Comments for improvement included individualized requests for specific content.

**Conclusions:**

The Carer Guide App was feasible and usable among carers of people with colorectal cancer. Acceptability can be improved through the inclusion of a variety of information and resources. A randomized controlled trial is required to assess the impact of the Carer Guide App on carers’ health and well-being.

## Introduction

In Australia, there are over 2.86 million informal carers who are not paid for the care they provide [1] and who often perform caring duties with limited training or guidance [2]. Many people with cancer rely on carers, such as family members or friends, for support during their illness trajectory [3] and for management of the side effects of treatment [3]. Carers looking after someone with cancer may experience unique needs related to their own health and well-being while in the caring period [4] and for as long as 5 years after the caring period [5].

A systematic review highlighted that Web-based interventions were feasible for use among cancer carers [6]; however, carers’ preference for information delivery varies across the disease trajectory. Smartphone apps can support carers by providing access to information, support, and resources from any place where an individual has internet connection [7]. Smartphone ownership continues to increase worldwide [8], creating an opportunity for apps to deliver health care content to large audiences [9]. Previous studies have assessed the use of smartphone apps among carers looking after a child with cancer [10] and looking after people with a variety of chronic illnesses [11]. Smartphone apps have been shown to improve participation in self-management of chronic illness [11], improve communication with health professionals [11], and promote detection of changes in cancer-related pain in children [10]. Further, several studies have evaluated the development of smartphone apps for carers of people with diabetes [12], adults looking after a child with cancer [13], and people living with back and spinal cord anomalies and their carers [14] and have described positive attitudes toward receiving support through smartphone apps. To our knowledge, no smartphone app has been trialed among carers looking after another adult with cancer [6]. In this study, we aimed to test the feasibility, usability, and acceptability of a smartphone app, called the Carer Guide App, in addressing the unmet needs of cancer carers.

## Methods

### Design

This study was a 30-day, single-arm pilot trial involving carers supporting people diagnosed with cancer. We chose colorectal cancer as it affects both men and women and is the third most common cancer worldwide [15,16].

We recruited carers of colorectal cancer patients from the second largest public health service and the largest not-for-profit private health service in Victoria, Australia. During 6 months, the public health service treated 105 people with colorectal cancer and the private health service had 273 admissions. Between October 2017 and May 2018, we approached carers during the patients’ chemotherapy appointment and provided them with an overview of the study. Patients nominated carers as being their main support person at home. Interested carers were provided with a recruitment pack (participant information sheet, consent form, and demographic questionnaire) to take home. When carers were unavailable at appointments, we approached patients, gave them an overview of the study, and asked whether their carer would be willing to participate. We asked the patients to take home the carer recruitment pack and sought initial consent via telephone to the carer within 48 hours to confirm participation. All carers provided written informed consent.

Adult carers of adult patients with colorectal cancer who were receiving chemotherapy or radiation treatment as day patients, either initial, recurrent, or secondary to surgery, were invited to participate. Carers were required to be in possession of a smartphone or tablet device and have internet access. At the end of the 30-day trial, carers received 2 reminder phone calls to return follow-up questionnaires. Follow-up occurred between November 2017 and May 2018. We obtained ethics approval from Deakin University (2017-218), Eastern Health (HREC/17/EH/24), and Epworth HealthCare (EH2016-169).

### Intervention

We developed the Carer Guide App using a codesign approach to address needs that carers identified in previous research [6,17,18]. A full description of the development process of the Carer Guide App is currently under review. The Carer Guide App was organized into 7 sections, each providing detailed information to address carers’ needs: Cancer Information, Carer Information, Well-being, My Social Network, Financial and Legal, Hospital Information, and Medical Terminology. In addition, two resources were provided: a Notepad and Contacts, which contained contact details for national information and support organizations and allowed carers to enter personal contact information. All carers had access to the Carer Guide App for 30 days and received 2 email messages each week directing them to information and services available within the app. Email messages related to carer health and well-being and support were available to carers. Messages were developed for each section of the Carer Guide App and provided information or reminders about the support that was available and where to locate this information within the Carer Guide App.

Upon enrollment in the study, carers provided a contact email address. We entered the nominated email address into the Carer Guide App system, which sent an automatically generated welcome email to carers. The welcome email included a link to download the Carer Guide App, a user identification number, password, and links to videos with instructions on how to download and navigate the Carer Guide App on both Android and iOS devices. Carers were provided with an email address to contact the research team for further technical support if required.

### Measures

#### Demographic Characteristics

We collected information on carers’ age, gender, living situation, relationship to the patient, level of education, and device type used for the study. Likert scales were developed to measure elements of feasibility, usability, and acceptability.

##### Feasibility

Feasibility included carers’ perception of the relevance of app content and accompanying email messages. We measured app content on a scale from extremely unuseful (1) to extremely useful (5). The helpfulness of email reminders was measured on a scale of extremely unhelpful (1) to extremely helpful (5). For the relevance of the content and usefulness of messages, carers could also respond with option 6, which represented “I did not use this icon” and “did not apply to me,” respectively. We developed Likert scales for the purpose of informing the relevance of each section of the Carer Guide App to inform future iterations. This follows guidelines where testing evaluation procedures can occur during feasibility studies [19]. A similar process has been used in the development of Web-based interventions, where scales have been validated during subsequent trials [20].

##### Usability

Usability included the navigation and readability of the app and was measured from strongly disagree (1) to strongly agree (5). We included open-ended questions to allow carers to provide comments for improving the Carer Guide app. We also measured usability by the number of people who accessed the instructional videos and who emailed the research team for technical support.

##### Acceptability

Acceptability included responses about carers’ use of the Carer Guide App for information and support, their desire to continue to use the app after the 30-day trial, and their feelings toward the Carer Guide App being made available to all carers. We measured items from strongly disagree (1) to strongly agree (5). App usage was measured quantitatively through self-reported usage and through Google Analytics records. Google Analytics tracked the number of log-ins, the duration of log-in, and the pages accessed.

### Statistical Analysis

We analyzed data using IBM SPSS (Version 25; IBM Corp). Feasibility, usability, and acceptability were analyzed by the frequency of agree (4) and strongly agree (5) responses. Demographic data and app usage from Google Analytics were analyzed using descriptive statistics.

## Results

### Demographic Characteristics

**Figure 1:**
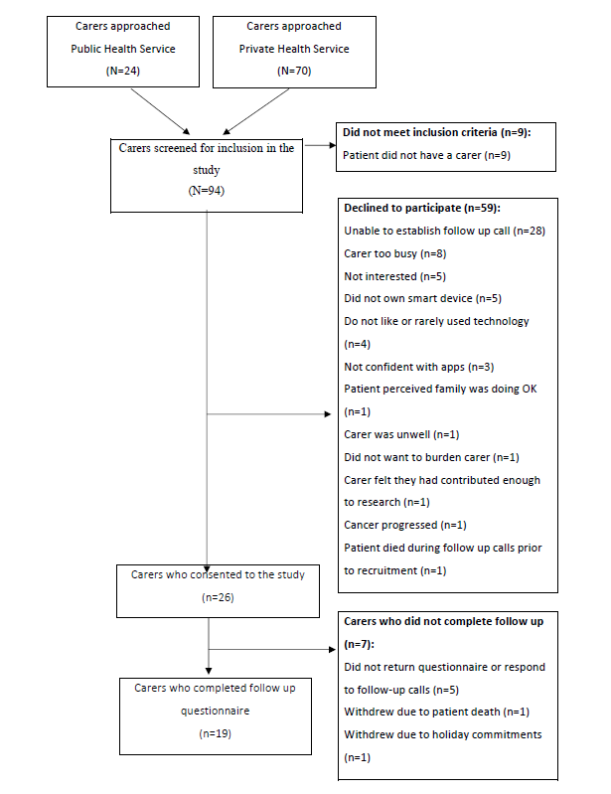
Flowchart of the recruitment process.

Of 85, a total of 26 (31%) carers consented to participate in the study, of which 20 (77%) used the Carer Guide App and 19 (73%) completed the follow-up questionnaires (attrition rate 7/26, 27%). Of the 7 carers who did not complete the follow-up questionnaire, 1 used the app and 6 did not use the app. There was 1 person who completed the follow-up questionnaire but did not use the app as he or she was not in need of it at the time. Figure 1 outlines the recruitment process.

The mean age of carers was 57 (SD 12; range 30-79) years. Of the 26 carers, the majority were female (19, 73%), caring for a spouse (26, 73%), and held a tertiary-level qualification (19, 73%); furthermore, the main device type used by carers was smartphones (15, 58%). There were 2 carers who accessed the Carer Guide App on their desktop computers (Web app version). Table 1 outlines the full demographic characteristics of the sample.

### Feasibility

#### Appropriateness of App Content

Of 19, the majority of carers rated Cancer Information (13, 68%), Carer Information (12, 63%), and Medical Terminology (12, 63%) as somewhat or extremely useful; the sections with the lowest agreement rate for usefulness were My Social Network (3, 16%) and Financial and Legal (4, 21%). Overall, the vast majority (16/19, 84%) of carers agreed or strongly agreed that the built-in links went to relevant websites. Table 2 provides detailed information on the usefulness of each section of the Carer Guide App.

#### Reminder Emails

Reminder emails were perceived as helpful by one-third of carers. The prompt to *take time out for yourself* related to well-being and was rated as helpful by the majority of carers (12/19, 63%), followed by Contact reminders for *information on holiday house programs* (10/19, 53%) and Carer Information *reminders to stay physically active* (10/19, 53%).

### Usability

The appearance and function of the Carer Guide App were reported as usable by the majority of carers; 17/19 (89%) found the font size appropriate, 13/19 (68%) found it easy to move between pages, and 11/19 (58%) stated that required information was easy to find. There were 4 carers who viewed the video instructions on how to download and navigate the Carer Guide App. The Carer Guide App’s email address was used by 7 carers to contact the research team with questions related to setting up the app on their phone or to provide comments in response to reminder emails sent.

### Acceptability

The majority of carers (16/19, 84%) agreed that the Carer Guide App should be available for all carers. Furthermore, 42% (8/19) stated that they used the app when they wanted more information and 42% (8/19) stated that they would like to continue using the app, while only 11% (2/19) reported that they used the app for support.

#### Usage

Findings from Google Analytics showed that over the 30-day trial period, a total of 71 log-ins occurred on the app. Of 19, more than one-third of carers (7, 37%) logged in 3-4 times during the 30 days and one-quarter (5, 26%) of carers logged in more than 5 times. On average, carers used the Carer Guide App twice (range 0-11), and median use was 17 minutes (interquartile range 4-35). Nearly half, 8/19 (42%) of carers used the Carer Guide app for longer than 30 minutes. According to self-reported usage, one-quarter (5/19, 26%) of carers used the Carer Guide App once a week. The top three most frequently used sections of the Carer Guide App were Cancer Information, Notepad, and Well-being, which were accessed 33, 33, and 31 times, respectively. Table 3 provides details of usage statistics.

**Table 1:** Demographic characteristics of the recruited carer participants (N=19).

Characteristics		Participants, n (%)	
**Device used**			
	Android mobile phone		5 (19)
	iOS mobile phone		10 (38)
	iOS tablet		3 (12)
	Computer desktop		2 (8)
**Gender**			
	Female		19 (73)
	Male		7 (26)
**Relationship status**			
	Spouse		19 (73)
	Other (parent, adult child, or sibling)		7 (27)
**Living with patient full time**			
	Yes		21 (81)
	No		5 (19)
**Education level**			
	Secondary		7 (27)
	Tertiary		19 (73)

**Table 2:** Carers’ responses of the usefulness of each section of the Carer Guide App (N=19).

App section	Median^a^	Agree or strongly agree responses (n=19), n (%)
Cancer Information	4	13 (68)
Carer Information	4	12 (63)
Medical Terminology	4	12 (63)
Well-being	4	9 (47)
Email messages	4	8 (42)
Contacts	3	8 (42)
Hospital Information	3	7 (37)
Notepad	3	6 (32)
Financial and Legal	3	4 (21)
My Social Network	3	3 (16)

^a^Results from a 5-point Likert scale (1=extremely unuseful to 5=extremely useful).

#### Qualitative Feedback

Of 19, 11 (58%) carers provided comments for improvements to the Carer Guide App. The majority of comments related to additions of items to the content, including the ability to journal events and symptoms, record a medical history and medical alerts, send to others for their use, and print off information sheets for the patient. Other comments related to content included more specific information about “red flags” for patients and carers; when to call the doctor; symptom information and management; medication information; contact details of doctors, nurses, and oncology wards; and information in different languages. Other comments included discrepancies in the tone of language used in medical terminology definitions, that the app should be delivered earlier in the caring period, and that the role of the carer needs to be more clearly highlighted, in particular, that the role varies across the illness trajectory.

Of all carers, 2 noted that the Carer Guide App gave them the confidence to deal with cancer-related issues and that it was the first time they felt someone cared about their needs.

**Table 3:** Carer Guide App usage including frequency of log-ins, duration of log-ins, and sections visited (N=19).

Characteristic		Value
**Frequency of log-ins (n=19), n (%)**		
	1-2 log-ins in 30 days	7 (37)
	3-4 log-ins in 30 days	7 (37)
	>5 log-ins in 30 days	5 (26)
**Duration of use per log-in (minutes)**		
	Mean (SD)	22 (21)
	Range	0-68
	Total duration of use over 30 days	403
**Total number of visits per Carer Guide App section (n=207), n (%)**		
	Cancer information	33 (16)
	Carer information	26 (13)
	Well-being	31 (15)
	My social network	19 (9)
	Financial and legal	16 (8)
	Contacts	14 (6)
	Hospital information	20 (10)
	Notepad	33 (16)
	Medical terminology	15 (7)

## Discussion

### Principal Findings

The Carer Guide App was developed in collaboration with carers to improve their access to information and support while looking after another adult with cancer. Overall, the Carer Guide App was a feasible option given the feedback received from participants. Carers perceived the content to be appropriate, and the links within the Carer Guide App led to relevant information. Certain sections of the app were perceived as more useful than others. However, all sections of the app received positive responses. These findings are comparable with Web-based interventions among people living with prostate cancer, where 47% of the people were satisfied with the program [21]. Differences in the perceived usefulness of the app sections may have several explanations. The type and amount of unmet needs experienced by carers constantly change [5]; therefore, a 30-day period to assess the appropriateness of content in addressing carers’ needs may not be long enough. Similarly, carers may experience different types of needs during different stages of the illness trajectory [22-25]. The static information provided in the Carer Guide App may not support needs as they evolve. Further, carers who had been in the carer role for a prolonged period may already have sourced the information and support required. It is possible that the Carer Guide App provided carers with information and resources that they were previously unaware of; however, more research is required to assess this. Despite these findings, 85% (16/26) of carers stated that the Carer Guide App should be available to all carers, and this is comparable to other studies evaluating the feasibility of cancer-related Web-based interventions [21].

Generally, carers found the email messages helpful in highlighting resources available within the Carer Guide App. Carers perceived the structure of the Carer Guide App as easy to navigate and locate information. Email support was used by several carers to enhance their experience and provide further instruction on using the app. These findings confirm the usefulness of technical support to aid the use of technology-based interventions for carers previously reported in the literature [6].

With a total of 71 log-ins and an average usage of 22 minutes over the 30-day trial period, the Carer Guide App was assessed to be acceptable to carers. Users often disengaged from sites within 10-20 seconds if they were unable to locate information [26]. As the average use in our sample was 22 minutes, this suggests that the Carer Guide App was acceptable for the information and resources provided within it. App usage varied greatly depending on the purpose of the app, and previous research has required participants to log in a specific number of times [10]. In another study involving a smartphone app providing static information for dementia, usage was on average 5 minutes for the duration of the 4-week period [27]. Further, findings of previous research suggested that smartphone apps did not impose a time burden on participants, and they could be incorporated into a daily routine from anywhere between 3 days to 1 year [10,11]. Carers reported that the Carer Guide App should be available to all carers, and suggestions for further improvements were mainly individual requests for specific information, resources, or design changes.

The recruitment rate of the study was modest (26/85, 31%); however, this is consistent with findings from previous research, where recruitment among this population can vary from 20% to 60% for technology-based intervention studies [6].

### Future Research Directions

Carers’ willingness to use smartphone apps and their need for this type of support may be impacted by patients’ stage of illness, carers’ knowledge of support available, and carers’ current support network. Future research may consider assessing smartphone app support at a certain stage of patients’ illness, for example, at diagnosis, to test its potential impact. This may provide information about the relevance of content to carers’ current and future needs, the ability of a smartphone app to meet needs, and carers’ likelihood of using a smartphone app during this stressful period. Future studies may also consider measuring carers’ knowledge of alternative support available and the presence and strength of their support network.

### Limitations

The sample was largely homogenous, with the majority of participants being female and highly educated, with all participants speaking English. In the general population in Australia, 31% has tertiary-level educational qualifications, [28] compared with 73% (19/26) of our sample, and 21% speak a language other than English at home [29]. Future studies should include larger samples to gain insights into feasibility, usability, and acceptability among a more heterogeneous sample. The duration of the caring period and patients’ stage of illness were not collected, which further limited the ability to determine whether the Carer Guide App was more feasible during specific stages of the caring or illness trajectory.

### Conclusions

A smartphone app may be appropriate for providing carers with more information and resources if the content is specific to their needs and provided at an optimal time during the caring period. The Carer Guide App is a feasible and acceptable method for delivering information and support to carers of people with colorectal cancer. Future iterations should include more specific information to enhance the acceptability of the App. Further research, including a randomized controlled trial, is recommended to assess whether a smartphone app has the potential to improve health and well-being outcomes and reduce unmet needs among carers.
